# HER2 Positive and HER2 Negative Classical Type Invasive Lobular Carcinomas: Comparison of Clinicopathologic Features

**DOI:** 10.3390/curroncol28030150

**Published:** 2021-04-24

**Authors:** Lin He, Ellen Araj, Yan Peng

**Affiliations:** 1 Department of Pathology, University of Texas Southwestern Medical Center, 6201 Harry Hines Blvd, Dallas, TX 75235, USA; lin.he@phhs.org (L.H.); Ellen.Araj@UTSouthwestern.edu (E.A.); 2 Harold C. Simmons Cancer Center, University of Texas Southwestern Medical Center, 5323 Harry Hines Blvd, Dallas, TX 75235, USA

**Keywords:** invasive lobular carcinoma, classical type, HER2, ER, Ki-67

## Abstract

Human epidermal growth factor receptor 2 (HER2) positive (+) classical type invasive lobular carcinoma (cILC) of the breast is extremely rare and its clinicopathologic features have not been well characterized. We compared features of HER2(+) and HER2 negative (−) cILCs. A total of 29 cases were identified from the clinical database at our institution from 2011-2019; 9 were HER2(+) cILC tumors and 20 were HER2(−) cILC tumors. The results reveal that HER2(+) cILC group had significantly increased Ki-67 expression and reduced estrogen receptor (ER) expression compared to HER2(−) cILC group (both *p* < 0.05). In addition, HER2(+) cILCs tended to be diagnosed at a younger age and more common in the left breast, and appeared to have a higher frequency of nodal or distant metastases. These clinicopathologic features suggest HER2(+) cILC tumors may have more aggressive behavior than their HER2(−) counterpart although both groups of tumors showed similar morphologic features. Future directions of the study: (1) To conduct a multi-institutional study with a larger case series of HER2(+) cILC to further characterize its clinicopathologic features; (2) to compare molecular profiles by next generation sequencing (NGS) assay between HER2(+) cILC and HER2(−) cILC cases to better understand tumor biology of this rare subset of HER2(+) breast cancer; and (3) to compare molecular characteristics of HER2(+) cILC and HER2(+) high grade breast cancer in conjunction with status of tumor response to anti-HER2 therapy to provide insight to management of this special type of low grade breast cancer to avoid unnecessary treatment and related toxicity

## 1. Introduction

Invasive lobular carcinoma (ILC) accounts for 3–15% of all invasive breast carcinomas [1,2,3,4,5,6,7,8]. The average age at diagnosis is around 55 to 60 [8,9] with some studies suggesting there are two peak age periods of risk for ILC, the first occurring in ages 40–50 years and the second after age 65 [2]. Foote and Stewart first described and classified lobular type breast carcinoma into in situ lesion [10] and invasive lesion [11]. The architectural pattern of “thread-like strands”, “loosely dispersed”, and “sheet-like” growth pretty much defines the classical type of ILC (cILC). Newman published the first large case series of lobular carcinoma in which he identified 142 of 1396 (10.1%) breast cancer cases to be terminal duct/lobular type [6]. Among these 142 tumors, he also found 73 tumors are “pure” lobular type (51.4%), i.e., classical type based on the exclusive “single cell” pattern. Another study reported 176 of 230 lobular carcinoma to be cILC (76.5%). E-cadherin is a member of transmembrane glycoproteins involved in Ca2^+^-dependent cell-cell adhesion. It acts as a tumor invasion suppressor gene and therefore has been associated with tumor morphogenesis. E-cadherin expression is largely lost (i.e., negative) in lobular carcinoma, which can be demonstrated by immunohistochemistry (IHC) and used to differentiate from other types of breast cancer, such as most commonly ductal carcinoma [12,13,14,15].

The first oncogenic receptor tyrosine kinase oncogene, neu, now also known as human epidermal growth factor receptor 2 (HER2) or c-erbB2 was discovered by transinfection and transformation of fragmented DNA from a series of rat neuroblastomas into NIH3T3 cells [16,17]. Slamon and colleagues found HER2/neu oncogene was amplified greater than 2- fold in 18% of the human primary breast cancers [18]. They also related the HER2/neu amplification to the survival probability and showed the prognostic value of HER2/neu in mammary [18] and ovarian malignancies [19]. The HER superfamily consists of four tyrosine kinase receptors: HER1 (epidermal growth factor receptor, EGFR), HER2 (neu, c-erbB2), HER3, and HER4 [20]. When activated, these receptors cause epithelial cell growth and differentiation. HER2 can be distinguished from HER1, 3, and 4 by differences in chromosomal location, transcript size, molecular mass, ligand activation of the associated tyrosine kinase, and antigenicity, as determined by interaction with specific monoclonal antibodies [21]. The HER2 oncogene encodes for a glycoprotein receptor with intracellular tyrosine kinase activity, and has no known ligand [22]. The other three HER receptors have known ligands, and form homodimers or heterodimers upon ligand binding, with the HER2 receptor being the preferred dimerization partner. The HER2 receptor can heterodimerize with the other receptors, which results in autophosphorylation of the tyrosine residues. This autophosphorylation subsequently activates the MAPK (mitogen-activated protein kinase) pathway and the PI3K (phosphatidylinositol 3-kinase) pathways [23]. With the discovery of HER2 target in breast cancer, anti-HER2 therapy, trastuzumab has significantly improved the survival of HER2-positive breast cancer patients for more than two decades.

The breast cancer is currently classified into four different molecular subtypes: Hormone receptor (HR)(+)/HER2(−) [Luminal A], HR(+)/HER2(+) [Luminal B], HR(−)/HER2(+) [HER2-enriched] and HR(−)/HER2(−) [triple negative or basal-like] [23,24,25,26,27]. Unlike invasive ductal carcinoma (IDC), cILCs are predominantly luminal A type [28,29,30]. ILCs with luminal B type or HER2-enriched type, both of which are HER2(+), often represent pleomorphic variant [29,31,32,33,34]. HER2(+) cILC is extremely rare with an estimated prevalence of 3–7% of all ILCs, therefore accounts for around 0.5% in all invasive carcinomas [24,28,30]. The clinicopathologic features of HER2 (+) cILC have not been well characterized, in particular when compared to HER2(−) cILC. The purpose of this study was to compare histopathologic features, prognostics and treatment between HER2(+) cILC and HER2(−) cILC groups along with review of the literature to provide insights to better understanding of tumor biology and prognosis of HER2(+) cILC, a rare subset of breast cancer.

## 2. Materials and Methods

A total of 29 patients with cILC were identified from the Epic Clarity Data Warehouse at University of Texas Southwestern Medical Center (UTSW, Dallas, TX, USA). The patients were identified by performing free text searches against all pathology reports from 2011–2020 using the Tableau Business Intelligence Tool (Tableau Software, LLC, Mountain View, CA, USA) at the UTSW university hospitals from 2011–2020. The lobular phenotype of these tumors was confirmed by the absence of E-cadherin immunostaining.

Immunohistochemical (IHC) stains were performed on an automated immunostainer (Ventana Benchmark XT) using Ventana primary antibodies. Scoring and quantification of ER, PR, Her2/neu, E-cadherin, and Ki-67 was performed on the most representative areas of the tumors.

HER2 IHC and FISH assessments followed the 2013 and 2018 guidelines for Her2 testing in breast cancer by American Society of Clinical Oncology (ASCO)/College of American Pathologists (CAP) [35,36]. HER2 IHC was scored on a scale from 0, 1+, 2+, and 3+. All cases with score of 3+ were defined as HER2 IHC positive and score of 0 or 1+ were HER2 negative; no HER2 FISH was performed on these cases. Only HER2 IHC equivocal (2+) cases were further evaluated by HER2 fluorescent in situ hybridization (FISH). HER2 FISH was scored as amplified if the ratio of the number of fluorescent signals of HER2 to chromosome 17 was ≥2.0 and average HER2 copy number/cell is >4 or if the ratio <2 and average HER2 copy number/cell is ≥6. According to the guidelines, indicators for anti-HER2 therapy are HER2 protein overexpression by IHC (score 3+) and/or HER2 gene amplification by FISH.

Statistical analysis was performed using R language (R Foundation, Vienna, Australia). Two-sample *t*-test was selected to compare continuous variables while chi-square test was selected to compare categorical variables. A significance level of 0.05 was used for the statistical tests.

## 3. Results

Nine patients had HER2(+) cILC and 20 had HER2(−) cILC. The mean age at diagnosis was 59.5 years (range: 41 to 69 years) in HER2(+) cILC group and 68.1 years (38 to 93 years) (*p* = 0.079, two-sample *t*-test). Regarding the laterality, 8 out of 9 (88.9%) HER2(+) cILC tumors and 11 of 20 (55.0%) HER2(−) cILCs were on the left breast (*p* = 0.076, chi-square test). Mean sizes of the tumor were similar between the two groups (3.4 cm vs. 3.7 cm). All of the tumors were Nottingham grade 1 or 2. Five of 9 patients with HER2(+) cILC (55.6%) had axillary lymph node metastases, while 4 of 19 patients with HER2(−) cILC (21%) had lymph node metastases and 2 of the 4 also had distant metastases. One patient with HER2(−) cILC was excluded from evaluation of status of nodal and distant metastasis because she did not receive surgery and/or systemic therapy due to her age (93 years-old) and other disease. All 29 patients were alive except two in the HER2(−) group died of medical complications at 38 and 44 months of diagnosis, respectively. The mean follow-up period was similar between these 2 groups, both of which were approximately 38 months.

ER was expressed in average 75.8% of the tumor cells in the HER2(+) group and 2 of the 9 cases only showed weak positivity. ER was expressed in nearly all tumor cells (average 99.2%) in the HER2(−) group and all of the cases showed strong positivity (*p* = 0.012, chi-square test). PR expression was also lower in the HER2(+) group than in the HER2(−) group (34.1% vs. 43.8%) but there was no statistical significance (*p* = 0.600, chi-square test). Three of 9 tumors in the HER2(+) group were HER2 IHC positive (3+), and the remaining 6 cases were HER2 IHC equivocal (2+) and reflex HER2 FISH positive. In the HER2(−) group, 19 of 20 tumors were HER2 IHC negative (0 or 1+); the remaining one was HER2 IHC 2+ and reflex FISH was negative (ratio of 1.38). The proliferation index Ki-67 was significantly higher in the HER2(+) group than that in the HER2(−) group (23.7% vs 9.3%, *p* = 0.009, chi-square test). 

Regarding the treatment, all patients but 2 in both groups underwent surgical resection. These 2 patients were in the HER2(−) group at age of 93 and 89 year-old respectively; one was redeemed inoperable due to the age and systemic conditions while the other had been treated with hormone treatment only. In the HER2(+) group, 3 of 9 patients (33.3%) received partial mastectomy; 2 of 9 patients (22.2%) received total mastectomy; and 4 of 9 patients (44.4%) received modified radical mastectomy. In the HER2(−) group, 8 of 20 patients (40.0%) received partial mastectomy; 3 of 20 patients (15.0%) received total mastectomy; and 7 of 20 patients (35.0%) received modified radical mastectomy.

All 9 patients (100%) in HER2(+) group received chemotherapy and trastuzumab (Herceptin) while only 7 of 20 patients (35%) in HER2(−) group had chemotherapy (*p* = 0.001). All patients in both groups except one in the HER2(−) group received hormone therapy. Approximately half of the patients in each group received radiation therapy after the surgery (55.6% vs. 50.0%, *p* = 0.593).

Table 1 summarizes the key clinicopathological features in HER2(+) and HER2(−) cILC groups.

**Figure 1:**
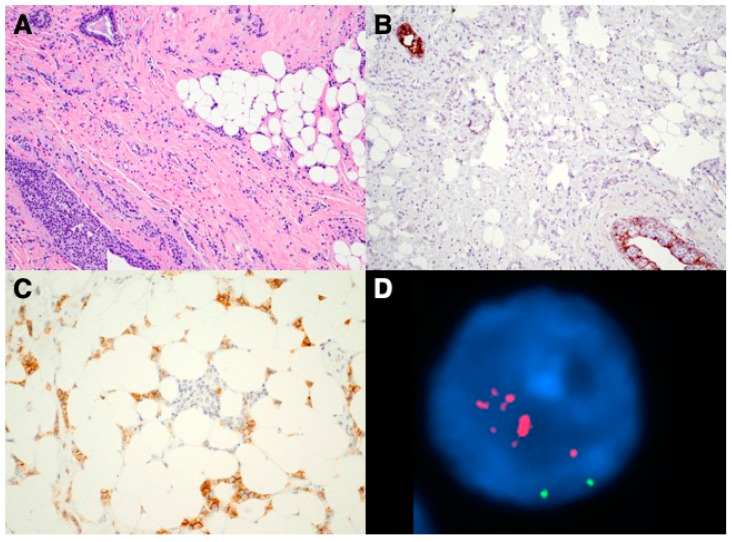
Images of a HER2(+) cILC case. (**A**) H&E section shows classic morphology of cILC with infiltrating small, uniform tumor cells present in single-file lines; (**B**) Immunostain confirms the complete loss of E-cadherin in the tumor cells (internal positive controls present); (**C**) HER2 IHC was interpreted as equivocal (2+); (**D**) Reflex FISH shows HER2 probe (in red fluorescence) was largely amplified (chromosome 17 centromere probe, in green fluorescence).

Figure 1 shows images from one of the HER2(+) cILC cases including morphology, immunohistochemistry and FISH. This is a 69 year-old woman who was diagnosed with cILC of the left breast on a biopsy. IHC stains showed ER and PR positive (100%, 15% respectively). HER2 by IHC was equivocal (2+) and reflex FISH was highly amplified with HER2/CEP17 ratio of 6.61 and average HER2 gene copy number/cell of 11.1. The patient underwent a left breast partial mastectomy and final resection pathology results confirmed to be a 2.5 cm cILC with repeat HER2 IHC and FISH were both positive. After the surgery, the patient received chemotherapy, trastuzumab and hormonal therapy, and completed radiation. 

## 4. Discussion

Unlike in the invasive ductal carcinoma of the breast with approximately 20% being HER2 amplified, previous studies have shown that HER2 is rarely overexpressed in the ILC especially in the classical type (cILC) which presents predominantly luminal A type [28,29,30,31,34,37,38]. HER2(+) cILC cases are extremely rare with only limited case reports in the literature. So far, there have been a couple of case series studies comparing HER2(+) and HER2(−) ILCs. The first study demonstrated HER2(+) ILC tends to present more aggressive pathological features including significantly less ER/PR expression compared to HER2(−) ILC [39]. The second study compared HER2(+) ILC and HER2(−) ILC cases and confirmed the findings in ER/PR expression [40]. However, both studies included pleomorphic type ILCs and/or mixed lobular and ductal invasive carcinomas; only 8 of 12 or 9 of 21 HER2(+) cases in each study were pure classical type (cILC). In our study, 9 HER2(+) and 20 HER2(−) pure cILC cases were included and more clinicopathologic features including nodal/distant metastases and treatment regimen were compared between the two groups of pure classical type of ILC although sample size in the HER2(+) group is relatively small.

Our study reveals that Ki-67 is significantly higher in the HER2(+) group than that in the HER2(−) group (*p* < 0.05), suggesting HER2(+) cILC may behave more aggressively in its clinical course [41], while the prior studies only showed higher Ki-67 in HER2(+) ILC group but without statistical significance [39,40]. In this study ER expression is significantly lower in the HER2(+) group while PR expression is lower than that in the HER2(−) group but the difference is not statistically significant. These findings are generally consistent with the prior studies [40,41,42,43] except that the PR expression in the HER2(−) ILC group seems to be reduced in our case series as well. 

In addition to the classic morphology of ILC, complete loss of E-cadherin further confirms the diagnosis of ILC. The classical type designation requires lack of pleomorphic features such as high-grade nuclear type, mixed features that include significant component of ductal structures, and rare intracytoplasmic mucin production [32,44]. These features have been shown to be associated with more aggressive tumor behavior and worse prognosis, which deviates from the typical clinical course of cILC. As a matter of fact, it is not uncommon that ILC can show more or less foci of ductal formation in the mixed type. Since prior studies also include non-classical type of ILC [39,40], this may bias the HER2(−) ILC group to be more proliferative versus the HER2(+) ILC group and therefore explain why Ki-67 was not statistically significant between the groups. Since HER2(+) cILC is extremely rare, a single-center study including our study and the prior two studies [39,40] hardly managed to collect more than 10 cases of HER2(+) cILC in each study. The limited case number in our study may affect the power of statistical analysis to show significant differences on other clinicopathological features such as frequency of nodal/distant metastases between two groups. A multicenter study in future to include more HER2(+) cILC cases will certainly be imperative to better characterize this rare breast cancer subtype.

One prior study [39] also subcategorize ILC morphology into histiocytoid [45,46,47], apocrine [24,48] and signet-ring variants [49,50] and found all 4 histiocytoid variant cases were in the HER2(+) group (33%). Similar findings have been demonstrated in other studies as well [24,51]. One of the limitations in our study is that the pathological reports retrieved from our database did not subcategorize ILC histiocytoid variant and therefore we could not confirm if histiocytoid variant was more predominant in the HER2(+) group. These morphological variants may have different tumor behaviors or prognostic indications. For example, apocrine differentiation is associated with androgen receptor expression which has been shown to result in better prognosis in triple-negative breast carcinoma [52]. Further workup may be helpful to understand the clinical relevance of these variants.

It is as expected that all patients in the HER2(+) group received chemotherapy in addition to trastuzumab, as a standard of care as compared to the HER2(−) group. Generally speaking, HER2(−) cILCs are not treated with chemotherapy unless patients are proven or suspicious for nodal or distant metastases. Anti-HER2 therapy and chemotherapy may help improve the overall survival in the HER2(+) group, that may explain similar overall survival between the two groups of cILC patients over the similar follow-up period in our study. It is also noted in general cILC has lower proliferative index (Ki-67) compared to ductal carcinoma regardless of the HER2 status. Low proliferative nature of the cILC may limit tumor response to chemotherapy and/or trastuzumab. In fact, in Zhang et al.’s study, it was reported that 3 of 21 HER2(+) ILC patients failed to achieve complete pathological response to neoadjuvant therapy [40]. Future studies appear to be essential to understand the overall survival benefit from anti-Her2 treatment on this subtype of morphologically low-grade tumors but with HER2 protein overexpression or HER2 gene amplification.

Discordance of HER2 status has been reported between primary tumor and the remaining tumor after chemotherapy in particular anti-HER2 treatment in more recent studies [28,53,54,55,56]. Intratumoral heterogeneity has been well documented in breast cancer especially among biopsy specimens [57]. In this study only one case showed discordant HER2 status; anti-HER2 therapy may play a role in loss of HER2 overexpression or it may represent an example of HER2 heterogeneity. Interestingly, this patient also had BCRA1 mutation, which is uncommon in ER/PR positive ILC with typical lobular histology. 

ILC is rare in other hereditary tumor syndromes and only accounts for a minority of cancers associated with well-established susceptibility genes, for example, comprising less than 10% of cancers in patients with BRCA2 mutations, and less than 5% of cancers in patients with BRCA1 or TP53 mutations [58]. BRCA1 mutation has also been found to be more commonly associated with basal-like or triple negative ILC [59]. E-cadherin, as a member of transmembrane glycoproteins involves in Ca2^+^-dependent cell-cell adhesion, acts as a tumor invasion suppressor gene. E-cadherin loss has been found to be associated with tumor characteristics including lobular histology, low grade, >2 cm in size, and HER2(−) breast cancer [60]. Its dysregulation results from somatic mutations in the CDH1 gene on chromosome 16q22.1, reported in 30% to 80% ILCs, as well as by loss of heterozygosity at the CDH2 locus [61,62]. In addition to CDH1 gene mutation, ILC and IDC differed in the FOXA1 and GATA3 mutational spectra, PTEN loss, and AKT1 activation and alterations in one of the three key genes of the phosphatidylinositol 3-kinase pathway, PIK3CA, PTEN, and AKT1, were present in more than one-half of the cases in the Cancer Genome Analysis study [63]. Of note, E-cadherin loss has been identified to be associated with AKT1 activation and EGFR overexpression [64,65]. Lack of E-cadherin expression, which characterizes almost all ILC tumors, may thus provide a favorable cellular context for AKT1 activation [63]. HER2 and HER3 were mutated in 5.1% and 3.6% of the tumors, both of which are involved in activating the human epidermal growth factor receptor pathway [66]. Further survival analyses in the same study revealed that chromosome 1q and 11p gains have independent prognostic value in ILC and that HER2 and AKT1 mutations were associated with increased risk of early relapse. The role of HER2 in relapsed ILC has also been suggested in another study [67]. Use of next generation sequencing (NGS) technique to analyze HER2(+) cILC samples can help to further characterize molecular features of this rare subset of low-grade breast cancer. By comparing their molecular features such as HER2 gene mutations and copy number alterations and p53 mutation status to those of HER2(+) high-grade breast cancer will demonstrate if there are any differences to better classify the HER2(+) cILCs to guide management of this rare subset of breast cancer to avoid unnecessary treatment and drug toxicity.

Outcome and prognosis in ILC are generally favorable especially in the luminal A type. In the SEER study including 27,639 patients with ILC and 235,769 patients with IDC treated between 1993 and 2003, a stage-matched analysis showed that the 5-year disease-free survival was significantly better for ILC than IDC with a hazard ratio of 0.86 [68]. However, due to its more indolent course, patients with ILC usually present at a higher stage at initial diagnosis [69], and a higher rate of late metastases in atypical locations compared to IDC [62]. Since HER2(+) breast cancer has been associated with poor prognosis and early metastasis, molecular studies have been conducted to demonstrate the underlying mechanism of HER2 in tumor metastasis. It has been shown that HER2 may cause E-cadherin downregulation [70], which subsequently causes tumor cells to become more discohesive and therefore increases the risk of metastasis. Harper et al. further identified a mechanism for early dissemination in which HER2 aberrantly activates a program similar to mammary ductal branching that generates early disseminated cancer cells that are capable of forming metastasis after a dormancy phase [71]. This mechanism may explain IDC metastasis in particular HER2(+) IDC metastasis well. However, for ILC that E-cadherin is already low or absent, other mechanisms seem to be necessary for its late atypical metastasis and warrant future studies. Our study has shown a higher metastatic rate in the HER2(+) ILC group but the overall survival is not statistically different from the HER2(−) ILC. This may be partly due to the efficacy of anti-HER2 treatment in HER2(+) group patients or relatively short follow-up period.

To understand the role of HER2 in ILC tumor biology at the molecular level is critical because there are more and more anti-HER2 drugs are being developed. Ever since trastuzumab, which binds to the domain IV region of the extracellular site of the HER2 protein has become a doorbuster in treating HER2(+) breast cancer by preventing dimerization, signal transduction and cell survival [72], newer drugs have been targeting combination of mutated genes to overcome drug resistance. Pertuzumab can prevent dimerization of HER1 (EGFR) and HER3 by binding to domain II of extracellular component of HER2. As a dual tyrosine kinase inhibitor, lapatinib binds to HER1 in addition to HER2 and therefore can subsequently prevents activation of MAPK pathway and AKT pathway. Similarly, neratinib inhibits HER1, HER2 and HER4 that can impact downstream signaling of MARPK and AKT pathways [63]. However, drug resistance to HER2 can develop if intracellular alteration occurs. ILC, in particular, has more frequent mutations in the PI3K pathway including PIK3CA, PTEN, and AKT1 [66], could potentially have a higher drug resistance rate. From medical oncology standpoint, it may be worthwhile to send HER2(+) cILC cases with uncertain prognosis potential to BluePrint, MammaPrint or Oncotype Dx test to obtain a more comprehensive molecular genetic picture of the tumor, that may help oncologists make treatment decision for management of this rare subset of HER2 (+) low grade breast cancer.

## 5. Conclusions

Our study reveals that HER2(+) cILC group had significantly increased Ki-67 expression and reduced ER expression compared to HER2(−) cILC group. The HER2(+) cILC tumors tended to be diagnosed at a younger age and more common in the left breast, and appeared to have a higher frequency of nodal or distant metastases. These clinicopathologic features suggest HER2(+) cILC tumors may behave more aggressively than their HER2(−) counterpart although both groups of tumors showed similar morphologic features. This case series study would expand the literature on this extremely rare subset of breast cancer. However, larger case series of HER2(+) cILC is needed to further characterize their tumor biology and prognosis.

## Figures and Tables

**Table 1:** Comparison of clinicopathologic features between HER2(+) and HER2(−) classical type invasive lobular carcinoma (cILC) groups (*p* < 0.05 highlighted in bold).

Clinicopathologic Features	HER2(+) cILC	HER2(−) cILC
Number of cases	9	20
Age at diagnosis	59.5 ± 3.1	68.1 ± 2.8
Follow-up time (month)	38.2 ± 8.2 (4–85)	38.4 ± 2.6 (24–74)
Laterality (left breast)	8 (87.5%)	11 (55.0%)
Tumor size (cm)	3.4 ± 1.0	3.7 ± 0.6
Nottingham grade	1.8 ± 0.1	1.6 ± 0.1
Nodal/distant metastasis	5 (55.6%)	5 (21.1%)
**ER expression**	**75.8 ± 14.6%**	**99.2 ± 0.4%**
PR expression	34.1 ± 14.0%	43.8 ± 10.1%
HER2 FISH ratio/gene copy #	3.5 ± 0.7 / 7.1 ± 1.3	Not performed
**Ki-67 expression**	**23.7 ± 6.8%**	**9.3 ± 1.6%**
**Chemotherapy**	**100% (+ Herceptin)**	**35%**
Hormone therapy	88.9%	100%
Radiation therapy	55.6%	50.0%

## Data Availability

Data is not available in the pulbic domain and therefore no links to publicly archived datasets are provided.
